# Probability of normal tissue complications for hematologic and gastrointestinal toxicity in postoperative whole pelvic radiotherapy for gynecologic malignancies using intensity-modulated proton therapy with robust optimization

**DOI:** 10.1093/jrr/rrae008

**Published:** 2024-03-17

**Authors:** Takaaki Yoshimura, Ryota Yamada, Rumiko Kinoshita, Taeko Matsuura, Takahiro Kanehira, Hiroshi Tamura, Kentaro Nishioka, Koichi Yasuda, Hiroshi Taguchi, Norio Katoh, Keiji Kobashi, Takayuki Hashimoto, Hidefumi Aoyama

**Affiliations:** Department of Health Sciences and Technology, Faculty of Health Sciences, Hokkaido University, Sapporo 060-0812, Japan; Department of Medical Physics, Hokkaido University Hospital, Sapporo 060-8648, Japan; Global Center for Biomedical Science and Engineering, Faculty of Medicine, Hokkaido University, Sapporo 060-8648, Japan; Department of Radiation Technology, Hokkaido University Hospital, Sapporo 060-8648, Japan; Department of Radiation Oncology, Hokkaido University Hospital, Sapporo 060-8648, Japan; Department of Medical Physics, Hokkaido University Hospital, Sapporo 060-8648, Japan; Faculty of Engineering, Hokkaido University, Sapporo 060–8638, Japan; Department of Medical Physics, Hokkaido University Hospital, Sapporo 060-8648, Japan; Department of Radiation Technology, Hokkaido University Hospital, Sapporo 060-8648, Japan; Global Center for Biomedical Science and Engineering, Faculty of Medicine, Hokkaido University, Sapporo 060-8648, Japan; Department of Radiation Oncology, Hokkaido University Hospital, Sapporo 060-8648, Japan; Department of Radiation Oncology, Hokkaido University Hospital, Sapporo 060-8648, Japan; Department of Radiation Oncology, Faculty of Medicine, Hokkaido University, Sapporo 060-8648, Japan; Global Center for Biomedical Science and Engineering, Faculty of Medicine, Hokkaido University, Sapporo 060-8648, Japan; Global Center for Biomedical Science and Engineering, Faculty of Medicine, Hokkaido University, Sapporo 060-8648, Japan; Department of Radiation Oncology, Faculty of Medicine, Hokkaido University, Sapporo 060-8648, Japan

**Keywords:** intensity-modulated proton therapy, robust optimization, retrospective treatment-planning study, normal tissue complication probability modeling analysis

## Abstract

This retrospective treatment-planning study was conducted to determine whether intensity-modulated proton therapy with robust optimization (ro-IMPT) reduces the risk of acute hematologic toxicity (H-T) and acute and late gastrointestinal toxicity (GI-T) in postoperative whole pelvic radiotherapy for gynecologic malignancies when compared with three-dimensional conformal radiation therapy (3D-CRT), intensity-modulated X-ray (IMXT) and single-field optimization proton beam (SFO-PBT) therapies. All plans were created for 13 gynecologic-malignancy patients. The prescribed dose was 45 GyE in 25 fractions for 95% planning target volume in 3D-CRT, IMXT and SFO-PBT plans and for 99% clinical target volume (CTV) in ro-IMPT plans. The normal tissue complication probability (NTCP) of each toxicity was used as an *in silico* surrogate marker. Median estimated NTCP values for acute H-T and acute and late GI-T were 0.20, 0.94 and 0.58 × 10^−1^ in 3D-CRT; 0.19, 0.65 and 0.24 × 10^−1^ in IMXT; 0.04, 0.74 and 0.19 × 10^−1^ in SFO-PBT; and 0.06, 0.66 and 0.15 × 10^−1^ in ro-IMPT, respectively. Compared with 3D-CRT and IMXT plans, the ro-IMPT plan demonstrated significant reduction in acute H-T and late GI-T. The risk of acute GI-T in ro-IMPT plan is equivalent with IMXT plan. The ro-IMPT plan demonstrated potential clinical benefits for reducing the risk of acute H-T and late GI-T in the treatment of gynecologic malignances by reducing the dose to the bone marrow and bowel bag while maintaining adequate dose coverage to the CTV. Our results indicated that ro-IMPT may reduce acute H-T and late GI-T risk with potentially improving outcomes for postoperative gynecologic-malignancy patients with concurrent chemotherapy.

## INTRODUCTION

Postoperative whole pelvic radiotherapy (WPRT) in gynecologic malignances has been recommended as an adjuvant treatment for patients who have one or more pathologic risk factors, including positive pelvic lymph nodes, parametrial extension and positive surgical margins [1]. WPRT is used to treat the pelvic lymph nodes, which lie on the right and left sides of the pelvis, as well as the apex of the vagina. During planning for WPRT, the potential impact on the surrounding critical structures must be considered to reduce acute and late adverse events. Although an acute adverse event can often be managed with medications and supportive care, late events may affect the patient’s quality of life (QOL) and sometimes may be life-threatening. The risk of major complications is related to the volume, total dose, dose per fraction and specific intrinsic radiosensitivity of the normal tissue that is irradiated. Therefore, it is important to minimize normal tissue exposure while maintaining tumor dose coverage for optimal outcomes.

Progress from use of the three-dimensional conformal radiation therapy technique (3D-CRT) to intensity-modulated X-ray therapy (IMXT), including volumetric arc therapy, has allowed increased conformity and significant dose reduction to organs at risk (OARs) [e.g. bone marrow (BM) and bowel bag (BB)], and this is associated with a lower incidence of adverse events such as acute hematologic toxicity (H-T) and acute or late gastrointestinal toxicity (GI-T) [2–4]. Increased incidence of acute H-T, including leukopenia, could limit tolerance for chemotherapy because of the larger irradiated volume of the pelvic BM, which is the primary site of hematopoiesis. Mell *et al.* have reported that an irradiated volume of pelvic BM receiving a dose of 10 and 20 Gy is associated with the incidence of acute H-T [5–7]. Additionally, for patients who have undergone hysterectomy, acute GI-T typically involves varying degrees of diarrhea, cramping and abdominal discomfort because of a larger irradiated volume of BB, which fills the pelvic cavity. Roeske *et al.* have indicated that the volume of BB irradiated with a dose of 45 Gy (${V}_{45 Gy\ \left(\mathrm{RBE}\right)}$) is a significant factor influencing the probability of acute GI-T occurrence [8]. To keep the incidence of acute GI-T within an acceptable range, quantitative analysis of normal tissue effects in the clinic recommended constraining ${V}_{45 Gy\ \left(\mathrm{RBE}\right)}$ to <195 mL [9]. Moreover, the incidence of late GI-T, such as obstruction, perforation or fistula, is uncommon, although this affects the patient QOL and sometimes leads to life-threating conditions. Therefore, it is important to reduce the incidence and severity of late GI-T as much as possible to improve patient QOL. Mundt *et al.* reported that IMXT for WPRT was associated with a significantly lower incidence rate of late GI-T than was 3D-CRT by reducing the dose to the BB [10].

Proton beam therapy (PBT) has a unique physical characteristic known as the Bragg-peak and offers the best sparing of OARs while maintaining an excellent target dose coverage when compared with IMXT. Recently, the spot-scanning proton therapy (SSPT) system has been used to provide a large treatment field (30 cm × 40 cm), which encompasses the entire pelvic region with one scanning field, and to enable the delivery of a complex dose distribution by magnetically scanned pencil beams for each spot and energy layer [11–14]. In several treatment planning studies of pelvic irradiation for gynecologic malignancies, compared with 3D-CRT and IMXT, intensity-modulated proton therapy (IMPT) using SSPT can reduce the irradiated volume of OARs [14–17].

There are two major optimization methods of dose calculation. One is single-field optimization (SFO) or the single-field uniform dose (SFUD), which allows each field to deliver the prescribed dose to the entire target volume and is less sensitive to uncertainties in patient setup and proton beam range [18, 19]. The other one is multifield optimization (MFO) in which all spots from all fields are optimized simultaneously. Although MFO produces more complex dose distributions than does SFO, MFO is more sensitive to uncertainties [20, 21]. Consequently, the planning robustness of MFO may be significantly degraded.

There are several published reports on robust optimization, which takes uncertainties into account during plan optimization [22–25]. For setup uncertainties, the isocenter of the patient is rigidly shifted in six directions. For range uncertainties, the stopping power ratios are modified by −3.5 and 3.5% corresponding to the maximum and minimum proton ranges, respectively [26, 27]. Thus, robust optimization is one of the most reasonable and effective strategies to accommodate uncertainties and has been introduced to commercial equipment.

For objective plan evaluation, the normal tissue complication probability (NTCP) based on dose-volume histograms (DVHs) is useful as an *in silico* surrogate marker to estimate the risk of adverse events for gynecologic malignancies [14,28–30]. In our previous report, compared with IMXT, SFO-PBT consisting of two opposite beams achieved significant reduction in the dose to the BM without compromising target coverage and risk of acute H-T based on the Lyman–Kutcher–Burman (LKB) NTCP model analysis [14]. However, there was no significant reduction in the dose to the BB between IMXT and SFO-PBT [14]. We therefore hypothesized that MFO-IMPT with robust optimization (ro-IMPT) reduces the dose to both the BM and BB. Consequently, the risk of H-T and GI-T in ro-IMPT would be lower than that in IMXT or SFO-PBT. This study aimed to compare the dose of ro-IMPT to that of 3D-CRT, IMXT and SFO-PBT and to evaluate the risk of H-T and GI-T using the LKB-NTCP model as an *in silico* surrogate endpoint.

## MATERIALS AND METHODS

### Patients

This retrospective dosimetric comparison study consisted of 13 patients who had previously received radiotherapy to the pelvic region for adjuvant treatment or recurrent diseases in gynecologic malignancies (eight cervical, four uterine and one ovarian) at our institution from 2008 to 2014. All patients had undergone hysterectomy (with or without pelvic lymph node dissection) and had received radiotherapy to pelvic region for adjuvant or recurrence after surgery. The body thickness was measured and defined as the umbilical cord level on treatment planning computed tomography (CT). This study was approved by the ethics committee of our hospital (019-0227). The requirement for written informed consent was waived owing to the retrospective nature of the study.

### Planning method

The CT that had acquired for photon radiotherapy was used for this planning study. The slice thickness were 2 or 2.5 mm. The details of our contouring strategy in this study have been stated in our previous report [14]. In short, all regions-of-interest were re-contoured uniformly delineated for this study using the Pinnacle^3^ treatment planning system (TPS) (ver.9.0; Philips, Inc, Madison, WI) according to the consensus guidelines [31, 32]. No changes were made due to clinical information.

As a reference, 3D-CRT plans with a four-field box arrangement were generated with a 10-MV photon beam and calculated using the Pinnacle^3^ TPS. Our IMXT and SFO-PBT treatment planning approach has been described in detail earlier [14]. In short, IMXT plans used seven evenly spaced intensity-modulated fields, which were generated with a 6-MV photon beam with a step and shoot multi leaf collimator technique and calculated using the Pinnacle^3^ TPS. Additionally, SFO-PBT plans consisted of an anterior–posterior (A-P) direction beam and were calculated using the VQA TPS (Hitachi, Ltd, Tokyo, Japan) assuming a PBT system, PROBEAT-RT (Hitachi, Ltd, Tokyo, Japan). All 3D-CRT, IMXT and SFO-PBT plans were designed such that 95% of the planning target volume (PTV), which was created by expanding the clinical target volume (CTV) with a 5-mm margin, received the prescribed dose. The prescribed dose was 45 Gy (RBE) in 25 fractions. Herein, RBE is the relative biological effectiveness value assumed generally to be 1.0 for photon beam therapy and 1.1 for PBT. The distal and proximal margins were beam-specific margins for expansion from the CTV during SFO-PBT [33].

In treatment planning for ro-IMPT, we used two posterior oblique fields to minimize the dose to the OARs and to maintain robustness against inter-fractional changes caused by factors like bowel gas or bladder filling, and calculated using the VQA TPS. Regarding ro-IMPT, plans were designed such that 99% of the CTV received the prescribed dose (CTV D99). The parameter for robust optimization was set at $\pm$3.5% for range uncertainties and 5 mm for setup uncertainties by shifting the patient’s CT image in the A-P, superior–inferior (S-I) and left–right (L-R) directions [26, 34]. The common dose constraints for OARs were bladder, ${V}_{45 Gy\ \left(\mathrm{RBE}\right)}$ < 35%; rectum, ${V}_{40 Gy\ \left(\mathrm{RBE}\right)}$ < 60%; BB, ${V}_{40 Gy\ \left(\mathrm{RBE}\right)}$ < 30%; femoral heads (FHs), ${V}_{30 Gy\ \left(\mathrm{RBE}\right)}$ < 15% and BM, ${V}_{10 Gy\ \left(\mathrm{RBE}\right)}$ < 90% and ${V}_{20 Gy\ \left(\mathrm{RBE}\right)}$ < 75% [14]. For robust evaluation of the CTV D99, we defined the non-shifted plan as the nominal plan and the six different plans with a 5-mm isocenter shift in the A-P, S-I and L-R directions as the robust plan.

### Data analysis

The DVH parameters were used to evaluate each plan (Table 2). For evaluation of the target coverage, the conformity index (CI) was defined as the ratio of the CTV covered by the prescribed dose and CTV, and the homogeneity index (HI) was defined as the ratio of the difference of D2 and D98 of the CTV and D50 of the CTV (the dose received by 2, 50 and 98% of the CTV, respectively) [35].

**Table 1:** Patient characteristics

		*n*	Median	Range		
				Min	–	Max
Age (year)		13	48	33		69
Primary site	Cervix	8				
	Uterine body	4				
	Ovary	1				
Height [m]		12	1.59	1.47	–	1.64
Weight [kg]		12	56.90	47.85	–	82.35
Body mass index (BMI) [kg/m^2^]		12	23.32	19.66	–	37.55
Body thickness [mm]		13	186.52	144.53	–	240.23
PTV volume [mL]		13	920.84	689.65	–	1041.79

**Table 2:** Dose constraints and summary of the DVHs analysis

			Constraints	(A) 3D-CRT plan (*n* = 13)				(B) IMXT plan (*n* = 13)			
				Median	Range			Median	Range		
					Min	–	Max		Min	–	Max
CTV	D99	[GyE]	> Prescribed dose	45.4	43.5	–	46.8	45.5	45.2	–	46.2
	CI			1.0	1.0	–	1.0	1.0	1.0	–	1.0
	HI			0.1	0.1	–	0.1	0.0	0.0	–	0.1
Bladder	V45	[%]	<35%	66.4	55.4	–	97.2	22.2	11.3	–	49.2
Rectum	V40	[%]	<60%	81.3	49.8	–	94.9	55.5	45.6	–	61.9
BB	V40	[%]	<30%	35.6	24.7	–	62.6	24.7	19.6	–	34.1
BM	V10	[%]	<90%	81.9	76.4	–	89.4	83.8	78.7	–	87.1
	V20	[%]	<75%	72.1	67.1	–	81.0	66.0	58.8	–	69.0
FH	V30	[%]	<15%	2.6	0.5	–	38.0	5.7	2.2	–	14.4
			Constraints	(C) SFO-PBT plan (*n* = 13)				(D) ro-IMPT plan (*n* = 13)			
				Median	Range			Median	Range		
					Min	–	Max		Min	–	Max
CTV	D99	[GyE]	> Prescribed dose	44.6	42.3	–	46.4	45.7	45.3	–	46.1
	CI			1.0	1.0	–	1.0	1.0	1.0	–	1.0
	HI			0.1	0.0	–	0.1	0.0	0.0	–	0.1
Bladder	V45	[%]	<35%	22.6	11.7	–	50.0	26.5	10.2	–	76.4
Rectum	V40	[%]	<60%	51.2	31.5	–	80.9	48.1	26.5	–	78.8
BB	V40	[%]	<30%	23.8	16.5	–	32.4	19.5	11.2	–	25.5
BM	V10	[%]	<90%	56.5	50.0	–	58.8	59.4	53.4	–	68.7
	V20	[%]	<75%	43.2	34.0	–	50.9	53.1	42.7	–	60.3
FH	V30	[%]	<15%	0.9	0.0	–	7.4	1.6	0.4	–	12.3

3D-CRT = three-dimensional conformal radiation therapy, IMXT = intensity-modulated X-ray therapy, SFO = single-field optimization, PBT = proton beam therapy, ro-IMPT = intensity-modulated proton therapy with robust optimization, CTV = clinical target volume, BB = bowel bag, BM = bone marrow, FH = femoral heads, CI = conformity index, and HI = homogeneity index.

We examined the plan robustness to assess the effect of uncertainties on the dose metrics for the CTV by computing the plan with a 5-mm isocenter shift in six directions (A-P, S-I and L-R) [13, 14, 34]. We evaluated acute or late GI-T by the volume of the BB receiving 5 to 45 GyE (${V}_{5 Gy\ \left(\mathrm{RBE}\right)}$ to ${V}_{45 Gy\ \left(\mathrm{RBE}\right)}$) in 5-GyE increments. Average DVHs for OARs were generated to display the combined DVH data for each plan.

For the estimation of the incidence of radiation-induced adverse events in WPRT, we used the Lyman–Kutcher–Burman normal tissue complication probability (LKB-NTCP) model with a generalized equivalent uniform dose (gEUD) for acute H-T and late GI-T as shown below [36–42]:


(1) \begin{equation*} {NTCP}_{LKB}=\frac{1}{\sqrt{2\pi }}{\int}_{-\infty}^t\exp \left(-\frac{x^2}{2}\right) dx \end{equation*}

(2) \begin{equation*} \mathrm{t}=\frac{gEUD-{TD}_{50}}{m\bullet{TD}_{50}} \end{equation*}

(3) \begin{equation*} gEUD={\left(\sum_{i=1}^k{v}_i{d_i}^{\frac{1}{n}}\right)}^n \end{equation*}

where ${TD}_{50}$ is the tolerance dose for a 50% complication probability for uniform doses to the organ, $m$ is the slope parameter, $n$ is the volume parameter, ${v}_i$ is the volume in a specific dose bin and $i$ and ${d}_i$ are the doses for each bin $i$ in differential DVHs. Moreover, we used a logistic NTCP function for acute GI-T, which correlated with dosimetric predictors as shown below [29]:


(4) \begin{equation*} {NTCP}_{log}=\frac{\exp \left(4\gamma \left(\frac{V}{V_{50}}-1\right)\right)}{1+\exp \left(4\gamma \left(\frac{V}{V_{50}}-1\right)\right)} \end{equation*}

where $V$ is the volume of the BB receiving a given dose level, ${V}_{50}$is the volume corresponding to a 50% incidence of complications, and $\gamma$ is the normalized slope of the volume-response curve. In this study, we used the following parameters: ${TD}_{50}=35\ \mathrm{GyE}$, $m=0.27$ and $\mathrm{n}=1$ for acute H-T [30]; ${V}_{50}=161\ \left[ mL\right]$ and $\gamma =0.31$ for acute GI-T [29] and ${TD}_{50}=55\ \mathrm{GyE}$, $m=0.16$ and $\mathrm{n}=0.15$ for late GI-T [40, 43]. The endpoints of these NTCP model parameters for acute H-T and acute GI-T were, respectively, grade $\ge$3 leukopenia, neutropenia or thrombocytopenia and grade $\ge$2 diarrhea, bleeding, abdominal pain or distension, based on the Common Terminology Criteria for Adverse Events, version 4.0. Similarly, the endpoints of the NTCP model parameters for late GI-T were obstruction, perforation or fistula.

For multiple comparisons, the Steel-Dwass test was used for all statistical comparisons between 3D-CRT, IMXT, SFO-PBT and ro-IMPT plans. Statistical significance was set at a *P*-value of <0.05. All statistical analyses were performed using JMP Pro ver. 16.2.0 (SAS Institute Inc, Cary, NC, USA).

## RESULTS

### Patients

The details of the patient characteristics were shown in Table 1. In one patient, there was no record of height and weigh on hospital information system because the outpatient treatment.

### Data analysis

**Fig. 1:**
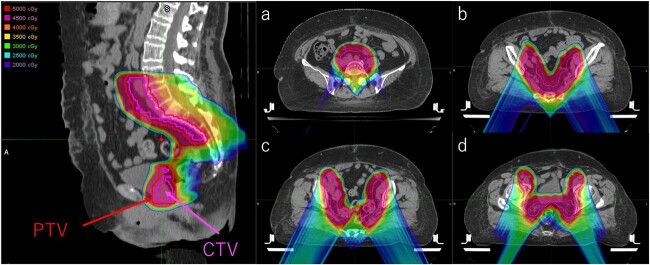
An example of dose distribution in ro-IMPT (Case No. 5). PTV = planning target volume, CTV = clinical target volume, (**a**) common iliac lymph nodes level, (**b**) presacral lymph nodes level (iso center), (**c**) obturator external iliac lymph nodes level, (**d**) paravaginal tissue level, ro-IMPT = intensity-modulated proton therapy with robust optimization.

**Fig. 2:**
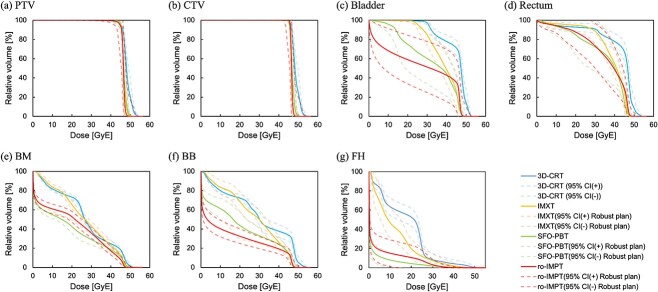
Average DVHs for PTV (**a**), CTV (**b**), bladder (**c**), rectum (**d**), BM (**e**), BB (**f**) and FH (**g**). The solid lines represent the average DVHs of the 13 patients for 3D-CRT, IMXT, SFO-PBT and ro-IMPT in the nominal plan. The surrounding dotted lines represent the 95% CIs for the robust plan. 3D-CRT = three-dimensional conformal radiation therapy technique, IMXT = intensity-modulated X-ray therapy, PBT = proton beam therapy, SFO = single-field optimization, ro-IMPT = intensity-modulated proton therapy with robust optimization, CTV = clinical target volume, PTV = planning target volume; BB = bowel bag, BM = bone marrow, FH = femoral heads; CI = confidence interval; DVHs = dose-volume histograms.

All plans achieved the prescribed dose to the CTV maintaining dose constraints in the clinically acceptable range. Figure 1 shows the dose distributions for the ro-IMPT plan. The dose distributions for IMXT and SFO-PBT have been reported in our previous study [14]. Figure 2 plots the DVH data for the PTV, CTV, bladder, rectum, BM, BB and FHs with their respective 95% confidence intervals in robust analysis. Dose-volume statistics for all patients are shown in Table 2 and Supplementary Material 1. There was no significant difference in the CTV D99 between the IMXT and ro-IMPT plans (*P* = 0.90). Although there were significant differences in HI for each plan, excellent dose conformity and homogeneity were observed in all plans. Moreover, the minimum dose in the worst case in the robust analysis, which was the 5-mm isocenter shifted in the A-P direction, under the ro-IMPT plan was 43.9 GyE.

There was no significant difference in the ${V}_{40 Gy\ \left(\mathrm{RBE}\right)}$ of BB reception between IMXT and SFO-PBT (*P* = 0.94). Conversely, there was a significant difference between the IMXT and ro-IMPT plans (*P* < 0.05). Moreover, there were significant differences in $the\ {V}_{30 Gy\ \left(\mathrm{RBE}\right)}$ of the FHs and ${V}_{10 Gy\ \left(\mathrm{RBE}\right)}$ and ${V}_{20 Gy\ \left(\mathrm{RBE}\right)}$ of the BM between the IMXT and ro-IMPT plans. Furthermore, there were no significant differences in other evaluation points in the bladder and rectum between each plan.

### Estimated risk of acute H-T, acute GI-T and late GI-T

The estimated risk of acute H-T in the ro-IMPT plan was sufficiently lower than that in the 3D-CRT and IMXT plans (*P* < 0.05) and slightly higher than that in the SFO-PBT plan (*P* < 0.05) but numerically lower enough. Although there was a significant difference between the estimated risk of acute GI-T in the 3D-CRT and other plans, as well as the SFO-PBT and IMXT or ro-IMPT plan, there were no significant differences between the IMXT and ro-IMPT plans. Although there was no significant difference between the SFO-PBT and ro-IMPT plans, the estimated risk of late GI-T in the ro-IMPT plan was significantly lower than that in the 3D-CRT and IMXT plans (*P* < 0.05).

**Fig. 3:**
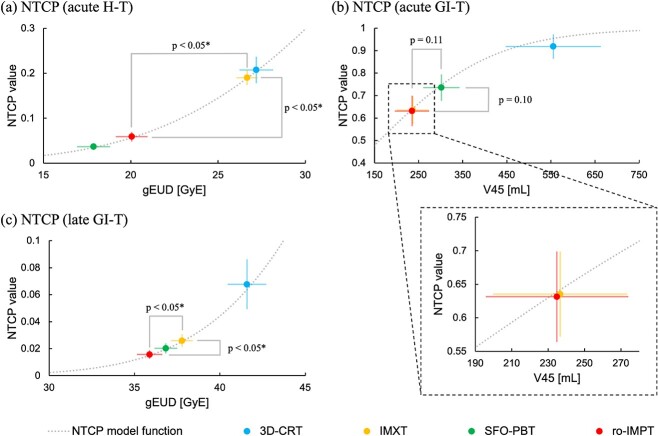
A scatter plot of estimated NTCP values for acute H-T (**a**), acute GI-T (**b**) and late GI-T in the ro-IMPT plan, compared with the values in the 3D-CRT, IMXT and SFO-PBT plans in our previous study. H-T = hematologic toxicity, GI-T = gastrointestinal toxicity, 3D-CRT = three-dimensional conformal radiation therapy technique, IMXT = intensity-modulated X-ray therapy, SFO = single-field optimization, PBT = proton beam therapy, ro-IMPT = intensity-modulated proton therapy with robust optimization, gEUD = generalized equivalent uniform dose, NTCP = normal tissue complication probability, and * = statistically significant difference (*P* < 0.05).

In the nominal plan, as shown in Table 3, Supplementary Material 2 and Fig. 3, the median and range of the gEUD and NTCP value for acute H-T in the ro-IMPT plan were significantly higher than those in the SFO-PBT plan but significantly lower than those in the 3D-CRT and IMXT plans (3D-CRT: gEUD = 27.2 GyE [24.7–30.0 GyE] and NTCP = 0.20 [0.14–0.30], IMXT: gEUD = 26.7 GyE [24.2–27.8 GyE] and NTCP = 0.19 [0.13–0.22], SFO-PBT: gEUD = 18.1 GyE [14.6–20.3 GyE] and NTCP = 0.04 [0.02–0.06], and ro-IMPT: gEUD = 20.1 GyE [16.3–22.4 GyE] and NTCP = 0.06 [0.02–0.09]). There was no significant difference in the ${V}_{45 Gy\ \left(\mathrm{RBE}\right)}$ of the BB and NTCP value for acute GI-T between the IMXT and ro-IMPT plans (IMXT: ${V}_{45 Gy\ \left(\mathrm{RBE}\right)}$ = 240.0 mL [140.6–353.5 mL] and NTCP = 0.65 [0.46–0.81], SFO-PBT: ${V}_{45 Gy\ \left(\mathrm{RBE}\right)}$ = 297.8 mL [195.8–414.5 mL] and NTCP = 0.74 [0.57–0.88], ro-IMPT: ${V}_{45 Gy\ \left(\mathrm{RBE}\right)}$ = 248.4 mL [141.0–347.8 mL] and NTCP = 0.66 [0.46–0.81]). Moreover, the gEUD and NTCP value for late GI-T in the ro-IMPT plan were significantly lower than those in the IMXT plan (IMXT: gEUD = 37.6 GyE [36.4–39.9 GyE] and NTCP = 0.24 × 10^−1^ [0.17 × 10^−1^-0.43 × 10^−1^], ro-IMPT: gEUD = 35.9 GyE [33.3–37.8 GyE] and NTCP = 0.15 × 10^−1^ [0.68 × 10^−2^-0.26 × 10^−1^]). Similar results were obtained in the robust evaluation. The ranges of the NTCP values for acute H-T, acute GI-T and late GI-T in robustness analysis were as follows: IMXT: 0.11–0.22, 0.38–0.89 and 0.14 × 10^−1^-0.54 × 10^−1^; SFO-PBT: 0.14 × 10^−1^-0.63 × 10^−1^, 0.56–0.89 and 0.10 × 10^−1^-0.32 × 10^−1^; and ro-IMPT: 0.22 × 10^−1^-0.13, 0.23–0.81 and 0.43 × 10^−2^-0.30 × 10^−1^.

**Table 3:** Estimated NTCP value of acute H-T, acute GI-T and late GI-T in nominal plan

	(A) 3D-CRT plan (*n* = 13)				(B) IMXT plan (*n* = 13)						
	Median	Range			Median	Range			*P* value		
		Min	–	Max		Min	–	Max	A-B	A-C	A-D
Acute H-T	0.20	0.14	–	0.30	0.19	0.13	–	0.22	0.87	<0.05	<0.05
Acute GI-T	0.94	0.67	–	1.00	0.65	0.46	–	0.81	<0.05	<0.05	<0.05
Late GI-T	$5.80\times{10}^{-2}$	$3.57\times{10}^{-2}$	–	$1.35\times{10}^{-2}$	$2.40\times{10}^{-2}$	$1.72\times{10}^{-2}$	–	$4.30\times{10}^{-2}$	<0.05	<0.05	<0.05
	(C) SFO-PBT plan (*n* = 13)					(D) ro-IMPT plan (*n* = 13)					
	Median	Range			Median	Range			*P* value		
		Min	–	Max		Min	–	Max	B-C	B-D	C-D
Acute H-T	0.04	0.02	–	0.06	0.06	0.02	–	0.09	<0.05	<0.05	<0.05
Acute GI-T	0.74	0.57	–	0.88	0.66	0.46	–	0.81	0.14	1.00	0.11
Late GI-T	$1.88\times{10}^{-2}$	$1.05\times{10}^{-2}$	–	$3.06\times{10}^{-2}$	$1.52\times{10}^{-2}$	$6.82\times{10}^{-3}$	–	$2.56\times{10}^{-2}$	0.51	<0.05	0.31

3D-CRT = three-dimensional conformal radiation therapy, IMXT = intensity-modulated X-ray therapy, SFO = single-field optimization, PBT = proton beam therapy, ro-IMPT = intensity-modulated proton therapy with robust optimization, H-T = hematologic toxicity, and GI-T = gastrointestinal toxicity.

## DISCUSSION

We estimated the risk of acute H-T, acute GI-T and late GI-T using the NTCP model as an *in silico* surrogate marker in the 3D-CRT, IMXT, SFO-PBT and ro-IMPT plans for postoperative WPRT (Fig. 3). The NTCP value for acute H-T in the ro-IMPT plan was found to be significantly higher than that in the SFO-PBT plan but sufficiently lower than that in the IMXT plan. The NTCP value for acute GI-T in the SFO-PBT plan with two opposite A-P direction beams was significantly higher than that in the IMXT plan. In contrast, there was no significant difference in the NTCP value for acute GI-T between the IMXT and ro-IMPT plans by avoiding the dose to the BB using two oblique posterior fields to minimize the dose to the OARs and to maintain robustness against inter-fractional changes caused by factors like bowel gas or bladder filling. Moreover, the NTCP value for late GI-T in the ro-IMPT plan was significantly lower than that in the IMXT plan. These results suggested that the risk of acute GI-T in the ro-IMPT plan was the same as that in the IMXT plan, although the ro-IMPT plan showed a reduced risk of acute H-T and late GI-T compared with the IMXT plan.

Previous studies have indicated that the use of IMXT for postoperative WPRT is associated with a reduced dose of OARs and toxicity compared with the use of 3D-CRT [2, 3, 44–47]. In this study, we estimated and evaluated not only the dosimetric parameters of OARs such as the BM and BB but also the risk of acute H-T, acute GI-T and late GI-T using the NTCP model. Although dosimetric comparison studies have indicated that the use of PBT for postoperative WPRT can reduce the dose to the BM and BB compared with the use of 3D-CRT and IMXT, to the best of our knowledge, this study is unique in predicting the risk of acute H-T, acute GI-T and late GI-T in the ro-IMPT plan using an NTCP model [13, 15, 17, 48]. Several studies have demonstrated that the volume of the BM receiving a low dose (${V}_{10 Gy\ \left(\mathrm{RBE}\right)}$, ${V}_{20 Gy\ \left(\mathrm{RBE}\right)}$) was associated with the incidence of acute H-T and the volume of the BB receiving a high dose (${V}_{45 Gy\ \left(\mathrm{RBE}\right)}$) was associated with the incidence of acute GI-T. As shown in the BM-sparing irradiation, the use of anterior or anterior oblique beams would be a natural choice [14, 15]. However, the use of anterior beams not only increases the irradiated volume of the BB but also strongly affects the plan robustness by inter-fractional changes by bowel gas or bladder filling. Our results demonstrated that ro-IMPT enabled a reduction in dose to the BM and BB without compromising CTV dose coverage relative to that for IMXT (Table 2).

The effect of uncertainties related to patient setup and inter-fractional anatomical changes are a critical issue in IMPT. In this study, $\pm$5 mm for setup uncertainties in all three dimensions was selected as a parameter of robust optimization. For the evaluation of CTV coverage, the CTV D99 was used for proton plan robustness [14, 47, 48]. Vyfhuis *et al.* reported that the clinically acceptable range of proton plan robustness in the worst-case scenario was >98% of the prescribed dose of the CTV [47]. As shown in Table 2, the CTV D99 in the worst-case scenario of the ro-IMPT plan was 97.6%. By utilizing orthogonal radiographs and cone-beam CT scans for daily patient setup, setup uncertainties could be reduced.

Currently, the use of PBT is increasing, although clinical experience with postoperative WPRT using PBT remains limited. In an initial report on the use of postoperative WPRT with PBT for gynecologic cancers, the incidence rate of acute H-T was 11% [13]. Bazan *et al.* reported that the incidence of acute H-T associated with postoperative WPRT using IMXT depends in both the radiation dose to the BM and chemotherapy regimen [30]. The rate of acute H-T associated with IMXT using chemotherapy was higher than that associated with radiation therapy alone. In our results, the NTCP value for acute H-T in the ro-IMPT plan was almost half that in the IMXT plan, similar to that reported in a previous study by Lin *et al.* [13]. These results suggested that it is possible to reduce the risk of acute H-T associated with concurrent chemoradiotherapy by PBT compared with IMXT. Although the NTCP value for acute GI-T in the SFO-PBT plan was significantly higher than that in the IMXT plan in our study, the NTCP value in the ro-IMPT plan was similar to that in the IMXT plan (Fig. 3). These results suggested that the incidence rate of acute GI-T in the ro-IMPT plan was equivalent to that in the IMXT plan. Moreover, we estimated the risk of late GI-T using the NTCP model based on the Emami parameters [40, 43]. Our results using the NTCP model as an *in silico* surrogate marker in the IMXT plan corresponded with those of previous clinical studies. Thus, our results showed that the ro-IMPT plan was associated with a lower risk of acute H-T and late GI-T than was the IMXT plan, although the risk of acute GI-T was equivalent to that in the IMXT plan.

This study has some limitations. First, we assumed the whole pelvic bone as the active BM, and we did not evaluate the functional BM. A multicenter phase II clinical trial of International Evaluation of Radiotherapy Technology Effectiveness in Cervical Cancer demonstrated that relative to 3D-CRT, IMXT reduced the risk of acute H-T and acute GI-T with promising therapeutic outcomes [49]. They also reported that functional BM-sparing IMXT with positron emission tomography reduced the incidence of acute H-T. Dinges *et al.* demonstrated that IMPT for cervical cancer reduced the dose to the functional BM in robust analysis under realistic systematic range uncertainties and clinically relevant setup errors [48].

Second, this study was a simulation study, rather than a report on the use of an actual treatment, including the uncertainty in NTCP modeling parameters. Although there have been numerous clinical trials comparing 3D-CRT and IMXT, limited clinical reports have compared IMXT with PBT, many of which were dose-comparative trials [13, 14, 17]. Further clinical trials comparing IMXT and PBT are required to confirm this. As shown in Fig. 3, we found that the NTCP value of acute H-T and late GI-T in the ro-IMPT plan was significantly lower than that in the IMXT plan, while the NTCP value of acute GI-T in the ro-IMPT plan was equivalent to that in the IMXT plan. It should be noted that the estimated NTCP values are typically subject to considerable model uncertainties. In this study, compared with the use of IMXT, that of ro-IMPT in postoperative WPRT for gynecologic malignancies reduced the risk of acute H-T and late GI-T without any risk of acute GI-T by significant reduction of the irradiated volume of the BM and BB. In particular, these results are beneficial for patients who require concurrent chemoradiotherapy for improved therapeutic outcomes. Reducing the risk of acute H-T may improve treatment outcomes by preventing chemotherapy interruption or discontinuation due to myelosuppression and maintaining treatment intensity. Similarly, since the late GI-T decreases not only patient QOL but can also be life-threatening in severe cases, the incidence rate reduction of late GI-T, such as intestinal perforation, may lead to maintenance of patient QOL and reduction of treatment-related death. Although our results were limited in a simulation study, we believe these results will provide the foundation for conducting future clinical trials.

Finally, since the planning robustness analysis in this study was limited by changes in the organ position, we cannot consider the planning robustness to be good enough based on inter-fractional changes in the intestinal tract or bladder capacity. Daily anatomical changes in the patient may affect the proton beam range uncertainties. For this problem, reliable volumetric imaging throughout the treatment session is needed. However, this planning robustness analysis could not be assessed in detail for the effect of anatomical changes because of the retrospective nature of the dosimetric simulation. Thus, a follow-up study based on the utilization of volumetric imaging such as daily cone-beam CT, online CT or magnetic resonance imaging is warranted.

## CONCLUSIONS

In this study, we evaluated the risk of acute H-T, acute GI-T and late GI-T in the 3D-CRT, IMXT, SFO-PBT and ro-IMPT plans using NTCP modeling analysis. The ro-IMPT plan demonstrated a significant reduction in the late GI-T risk. Moreover, although the risk of H-T in the ro-IMPT plan was higher than that in the SFO-PBT plan, the risk of H-T in the ro-IMPT plan was significantly lower than that in the 3D-CRT and IMXT plans. Thus, the ro-IMPT plan demonstrated potential clinical benefits for reducing the risk of acute H-T and late GI-T in the treatment of gynecologic malignances by reducing the dose to the BM and BB while maintaining adequate dose coverage to the CTV. Although this was an *in silico* study, our results indicated that compared with IMXT, ro-IMPT has the potential to reduce the risk of acute H-T and late GI-T, which may lead to improved outcomes for patients receiving concurrent chemotherapy for postoperative gynecologic malignancies.

## Supplementary Material

Supplementary_Material_1_20231221_rrae008

Supplementary_Material_2_20231221_rrae008

## Previous Presentations

A preliminary version of this study was presented at the 33rd Annual Meeting of the Japanese Society for Radiation Oncology (JASTRO), 1–3 October 2020, and the 62nd Annual Meeting of the American Society of Radiation Oncology (ASTRO), 25–28 October 2020.
