# Supplementary material for: Probability of normal tissue complications for hematologic and gastrointestinal toxicity in postoperative whole pelvic radiotherapy for gynecologic malignancies using intensity-modulated proton therapy with robust optimization
Source: J Radiat Res. 2024 Mar 17;65(3):369–78. doi: 10.1093/jrr/rrae008 (PMC11115445; doi:10.1093/jrr/rrae008)
Supplement: Supplementary_Material_1_20231221_rrae008 [file supplementary_material_1_20231221_rrae008.docx]

**Supplementary Material 1** Dose constraints and summary of the DVHs analysis. 3D-CRT: three-dimensional conformal radiation therapy, IMXT: intensity modulated X-ray therapy, PBT: proton beam therapy, SFO: single field optimization, ro-IMPT: intensity modulated proton therapy with robust optimization, CTV: clinical target volume, BB: bowel bag, FH: femoral heads, BM: bone marrow, CI: conformity index, and HI: homogeneity index.

|  | | | Constraints | A) 4-field box plan (n=13) | | | | B) IMXT plan (n=13) | | | | C) SFO-PBT plan (n=13) | | | | | D) ro-IMPT plan (n=13) | | | | |
| --- | --- | --- | --- | --- | --- | --- | --- | --- | --- | --- | --- | --- | --- | --- | --- | --- | --- | --- | --- | --- | --- |
|  |  |  |  | Median | Range | | | Median | Range | | | Median | Range | | | | Median | Range | | | |
|  |  |  |  |  | Min | - | Max |  | Min | - | Max |  | Min | - | Max |  | | Min | - | Max |  |
| PTV | D95 | [GyE] | > Prescribed dose | 45.6 | 42.2 | - | 46.1 | 95.2 | 94.9 | - | 95.5 | 95.2 | 95.0 | - | 95.6 | 94.4 | | 90.1 | - | 98.1 |  |
| CTV | D99 | [GyE] | > Prescribed dose | 45.4 | 43.5 | - | 46.8 | 45.5 | 45.2 | - | 46.2 | 44.6 | 42.3 | - | 46.4 | 45.7 | | 45.3 | - | 46.1 |  |
|  | CI |  |  | 1.0 | 1.0 | - | 1.0 | 1.0 | 1.0 | - | 1.0 | 1.0 | 1.0 | - | 1.0 | 1.0 | | 1.0 | - | 1.0 |  |
|  | HI |  |  | 0.1 | 0.1 | - | 0.1 | 0.0 | 0.0 | - | 0.1 | 0.1 | 0.0 | - | 0.1 | 0.0 | | 0.0 | - | 0.1 |  |
| Bladder | V45 | [%] | <35% | 66.4 | 55.4 | - | 97.2 | 22.2 | 11.3 | - | 49.2 | 22.6 | 11.7 | - | 50.0 | 26.5 | | 10.2 | - | 76.4 |  |
| Rectum | V40 | [%] | <60% | 81.3 | 49.8 | - | 94.9 | 55.5 | 45.6 | - | 61.9 | 51.2 | 31.5 | - | 80.9 | 48.1 | | 26.5 | - | 78.8 |  |
| BB | V5 | [mL] |  | 1426.5 | 939.3 | - | 2129.1 | 1539.6 | 920.3 | - | 2243.5 | 1172.5 | 645.2 | - | 1589.1 | 734.7 | | 533.8 | - | 1020.9 |  |
|  | V10 | [mL] |  | 1264.2 | 829.2 | - | 1887.1 | 1414.0 | 797.5 | - | 2020.5 | 1089.8 | 593.1 | - | 1274.5 | 644.4 | | 462.3 | - | 897.3 |  |
|  | V15 | [mL] |  | 1173.8 | 756.7 | - | 1772.0 | 1341.4 | 737.3 | - | 1917.9 | 953.9 | 514.7 | - | 1112.4 | 583.9 | | 417.7 | - | 816.2 |  |
|  | V20 | [mL] |  | 1129.4 | 715.0 | - | 1702.1 | 1209.0 | 624.9 | - | 1616.3 | 718.5 | 444.8 | - | 1060.2 | 535.6 | | 385.2 | - | 755.4 |  |
|  | V25 | [mL] |  | 1043.2 | 617.6 | - | 1517.5 | 1038.2 | 516.5 | - | 1288.4 | 609.0 | 391.5 | - | 820.5 | 480.6 | | 334.0 | - | 691.4 |  |
|  | V30 | [mL] |  | 859.5 | 396.9 | - | 1228.8 | 893.1 | 448.7 | - | 1087.1 | 539.0 | 346.4 | - | 720.7 | 431.7 | | 288.6 | - | 622.4 |  |
|  | V35 | [mL] |  | 618.1 | 310.9 | - | 1109.2 | 653.8 | 337.9 | - | 797.4 | 471.6 | 301.7 | - | 630.2 | 381.5 | | 246.0 | - | 547.2 |  |
|  | V40 | [%] | <30% | 35.6 | 24.7 | - | 62.6 | 24.7 | 19.6 | - | 34.1 | 23.8 | 16.5 | - | 32.4 | 19.5 | | 11.2 | - | 25.5 |  |
|  | V40 | [mL] |  | 568.0 | 277.6 | - | 992.7 | 459.7 | 247.1 | - | 553.3 | 393.4 | 250.5 | - | 522.2 | 330.2 | | 205.4 | - | 470.9 |  |
|  | V45 | [mL] |  | 497.8 | 243.0 | - | 839.1 | 240.0 | 140.6 | - | 353.5 | 235.7 | 161.6 | - | 324.5 | 248.4 | | 141.0 | - | 347.8 |  |
| FH | V30 | [%] | <15% | 2.6 | 0.5 | - | 38.0 | 5.7 | 2.2 | - | 14.4 | 0.9 | 0.0 | - | 7.4 | 1.6 | | 0.4 | - | 12.3 |  |
| BM | V10 | [%] | <90% | 81.9 | 76.4 | - | 89.4 | 83.8 | 78.7 | - | 87.1 | 56.5 | 50.0 | - | 58.8 | 59.4 | | 53.4 | - | 68.7 |  |
|  | V20 | [%] | <75% | 72.1 | 67.1 | - | 81.0 | 66.0 | 58.8 | - | 69.0 | 43.2 | 34.0 | - | 50.9 | 53.1 | | 42.7 | - | 60.3 |  |
