# Supplementary material for: Probability of normal tissue complications for hematologic and gastrointestinal toxicity in postoperative whole pelvic radiotherapy for gynecologic malignancies using intensity-modulated proton therapy with robust optimization
Source: J Radiat Res. 2024 Mar 17;65(3):369–78. doi: 10.1093/jrr/rrae008 (PMC11115445; doi:10.1093/jrr/rrae008)
Supplement: Supplementary_Material_2_20231221_rrae008 [file supplementary_material_2_20231221_rrae008.docx]

**Supplementary Material 2** Estimated NTCP value and dosimetric parameter (gEUD of BM and BB, or $V_{45Gy (RBE)}$ of BB) of acute H-T, acute GI-T, and late GI-T in nominal plan and robust plan. BB: bowel bag, BM: bone marrow, 3D-CRT: three-dimensional conformal radiation therapy, IMXT: intensity modulated X-ray therapy, PBT: proton beam therapy, SFO: single field optimization, ro-IMPT: intensity modulated proton therapy with robust optimization, H-T: hematologic toxicity, GI-T: gastrointestinal toxicity, gEUD: generalized equivalent uniform dose, and NTCP: normal tissue complication probability.

|  | | A) 3D-CRT plan (n=13) | | | | B) IMXT plan (n=13) | | | |
| --- | --- | --- | --- | --- | --- | --- | --- | --- | --- |
|  |  | Median | Range | | | Median | Range | | |
|  |  |  | Min | - | Max |  | Min | - | Max |
| Acute H-T | gEUD [GyE] | 27.18 | 24.68 | - | 30.05 | 26.69 | 24.17 | - | 27.77 |
|  | NTCP Value | 0.20 | 0.14 | - | 0.30 | 0.19 | 0.13 | - | 0.22 |
| Acute GI-T | V45 [mL] | 512.58 | 251.85 | - | 869.82 | 240.00 | 140.58 | - | 353.47 |
|  | NTCP Value | 0.94 | 0.67 | - | 1.00 | 0.65 | 0.46 | - | 0.81 |
| Late GI-T | gEUD [GyE] | 41.17 | 39.13 | - | 45.30 | 37.61 | 36.39 | - | 39.89 |
|  | NTCP Value | ${5.80\times10}^{-2}$ | ${3.57\times10}^{-2}$ | - | ${1.35\times10}^{-2}$ | ${2.40\times10}^{-2}$ | ${1.72\times10}^{-2}$ | - | ${4.30\times10}^{-2}$ |
|  | | C) SFO-PBT plan (n=13) | | | | D) ro-IMPT plan (n=13) | | | |
|  |  | Median | Range | | | Median | Range | | |
|  |  |  | Min | - | Max |  | Min | - | Max |
| Acute H-T | gEUD [GyE] | 18.09 | 14.55 | - | 20.28 | 20.07 | 16.32 | - | 22.40 |
|  | NTCP Value | 0.04 | 0.02 | - | 0.06 | 0.06 | 0.02 | - | 0.09 |
| Acute GI-T | V45 [mL] | 297.80 | 195.83 | - | 414.48 | 248.39 | 140.98 | - | 347.79 |
|  | NTCP Value | 0.74 | 0.57 | - | 0.88 | 0.66 | 0.46 | - | 0.81 |
| Late GI-T | gEUD [GyE] | 36.70 | 34.69 | - | 38.53 | 35.95 | 33.29 | - | 37.84 |
|  | NTCP Value | ${1.88\times10}^{-2}$ | ${1.05\times10}^{-2}$ | - | ${3.06\times10}^{-2}$ | ${1.52\times10}^{-2}$ | ${6.82\times10}^{-3}$ | - | ${2.56\times10}^{-2}$ |
